# Abnormal anus and hymen in a toddler: A case report

**DOI:** 10.1177/2050313X251336057

**Published:** 2025-04-24

**Authors:** Nancy D. Kellogg, Lora R. Spiller

**Affiliations:** 1 Division of Child Abuse, Department of Pediatrics, University of Texas Health Science Center at San Antonio, TX, USA; 2 Center for Miracles, CHRISTUS Children’s, TX, USA

**Keywords:** child sexual abuse, congenital malformation, ectopic anus, perineal fistula

## Abstract

A 2-year-old girl presented to the Emergency Department with genital complaints and a concern for sexual abuse. A perineal defect and absence of a segment of the posterior hymen were observed; the caregiver denied a past history of trauma. No other signs consistent with abuse or neglect were noted on examination. Pediatric surgery conducted an exam under anesthesia and confirmed a perineal fistula. While trauma is the most likely explanation for a large segmental defect in the posterior hymen, the co-occurrence of an anatomical variant of the anus and perineum in this patient raises questions about the etiology of both findings. This case underscores the importance of adequate examinations with careful inspection and documentation of anogenital structures in the newborn period.

## Introduction

Posterior hymenal transections or defects are considered indicative of penetrating trauma and, in children, are generally attributed to sexual assault.^1^ While studies have described anatomical variants in newborn infants, the absence of the hymen and complete posterior hymenal defects have not been described in newborns.

Anorectal malformations occur in ~1/5000 births. A perineal fistula is a type of anorectal malformation that has been previously described as an anteriorly located anus that is not completely surrounded by the sphincter muscle complex.^2^ About 70% of anorectal malformations are associated with other congenital anomalies, including cardiac, gastrointestinal, musculoskeletal, spinal cord, and urogenital anomalies.^3^ When detected at birth, further evaluation to identify fistulas and to establish the need for a colostomy is indicated.^4^ In addition, consultation with a genetics specialist and a comprehensive assessment for other congenital anomalies should be conducted following birth.

The diagnosis of sexual abuse or assault in children relies primarily on the history of the child, as findings of trauma are typically absent. In the absence of a witness or confession, the diagnosis of sexual abuse in a preverbal child is challenging as it relies on other evidence, such as a witness, a sexually transmitted disease, acute or healed trauma, or recovery of foreign DNA, which is rarely recovered in young children.

We report a case of a 2-year-old preverbal girl who presented with a complete posterior hymenal defect and a perineal fistula. The case is presented according to the ethical framework of the institution and consent of the parent was received for publication.

## Case report

A 2-year-old girl presented to the Emergency Department with a female family friend who had been caring for the child for about 2 weeks. She presented due to diaper rash, cough, and congestion. The caregiver indicated that after a recent 2-day visit with a relative, the child would point to her genitals and say “ow.” The caregiver also commented on the unusual appearance of her genitals. She was referred to a sexual assault nurse examiner who documented a healed posterior hymenal transection.

She had a previous Emergency Department visit for “diaper rash” at age 7 months; no abnormal anogenital findings were documented for that visit. Immunization records indicate that the child received only one set of vaccines at age 5 months. Documentation was limited, but no prior medical problems or birth anomalies were noted. The caregiver denied knowledge of past trauma and the concern for an abnormal genital exam was reported to the child protection agency, resulting in an investigation for sexual abuse.

One month later, the girl and her mother presented for a follow-up examination. The mother stated that since birth the girl’s genitals “looked different” from her other daughters’, with “one open hole instead of the three she’s supposed to have.” Mother reported frequent loose stools and diarrhea and having to “clean out her vagina.” The girl also had frequent anogenital rashes. The child wore diapers and the mother denied any previous history of anogenital bleeding or trauma. Social history was significant for an incarcerated parent and 12-year-old sister who was a victim of sex trafficking.

**Figure 1.**
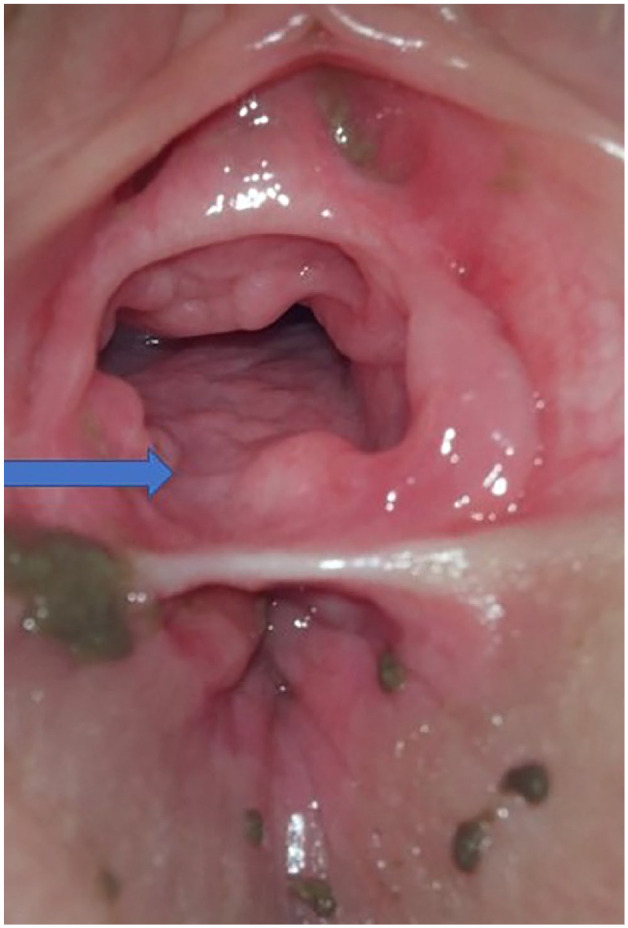
Two-year-old child in supine position with anteriorly displaced anus and a narrow white band of horizontal tissue at the posterior commissure, separating the vagina and anal mucosa. No hymen is observed between 6 and 8 o’clock (arrow).

General examination, including an assessment for physical abuse and neglect, was normal and her development was appropriate for her age. On examination with a photo colposcope, the girl had an anteriorly positioned anus with a narrow white band of horizontal tissue at the posterior commissure, separating the vaginal and anal mucosa. No hymen was observed between 6 and 8 o’clock (Figure 1). The anus was noted to have a poor tone. Examination of the child in a prone knee-chest position confirmed the complete hymenal defect from 6 to 8 o’clock.

She was referred to pediatric surgery, which conducted an evaluation under anesthesia. The urethra was normal, and a minute perineal body was observed. Per surgical report, the anus was centered within the muscle complex and there was a defect in the anterior segment; no anogenital scarring was observed. Also noted was the absence of a segment of the posterior hymen. Postoperative diagnosis was a perineal fistula.

Efforts to locate the family after the surgical examination were unsuccessful. Another referral for concerns of medical neglect was made due to several missed appointments.

More than 2 years later, she presented for a foster care initial examination in the same healthcare system; she was placed in foster care due to concerns of neglect/insecure housing. “Normal prepubertal genitalia” was documented. She had not made a disclosure of sexual abuse. There were reports of infrequent urinary and fecal incontinence. Her anogenital examination at age 5 was confirmed to be unchanged from the initial Emergency Department/Sexual Assault examination. No abnormalities of the vertebra, trachea, esophagus, or limbs were identified on initial or subsequent examinations. Her caregivers were provided with written documentation describing the girl’s anatomy to avoid future concerns for sexual abuse based solely on her abnormal anogenital exam findings.

## Discussion

This child presented with a large posterior hymenal defect, which is considered indicative of penetrating trauma. The hymenal finding was associated with an anatomical variant of the rectum and perineum, raising the question of whether one etiology could explain both findings. Her findings, along with the lack of known trauma, do not exclude or support a traumatic etiology. Known congenital variations of the hymen include imperforate hymen and anterior hymenal clefts, but congenital absence of the hymen in the posterior rim segment has not been previously described.^5,6^ In this child, penetrating trauma, either because of sexual assault or as a result of mother “going inside and cleaning poop out of her vagina” are plausible explanations for the hymenal findings, but do not explain the perineal and rectal findings. In this case, the parent indicates the genitals appeared different from birth when compared with her other daughters, but there was no medical documentation until the child was 2 years old. In addition, no abnormalities were observed or documented in a subsequent examination, emphasizing the need for adequate inspection and documentation of anogenital structures in the newborn period.

When the anal opening is <10% of the distance from the fourchette to the coccyx, the anal sphincter may not completely encircle the opening, resulting in stool leakage, as seen in this child at the time of her initial examination. After the evaluation by surgery, the child was lost to follow-up so further evaluation by genetics and for other congenital anomalies was not completed at that time; however, on follow-up examination, no other congenital abnormalities were identified.

**Figure 2.**
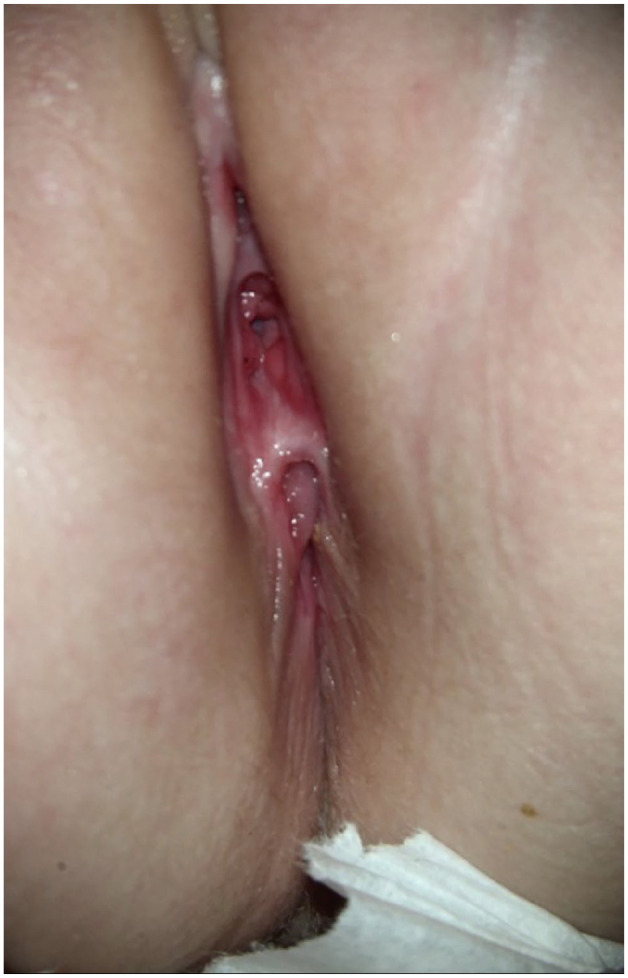
The anatomical defect is readily visible when the child is placed in a frog leg position without any traction on the genital tissues.

An ectopic anus/perineum was first described in 1955, and while rare, has not been associated with hymenal defects.^7^ Of note in this case, the child presented as a 5-month-old infant with a genital complaint and while a rash was observed, no anatomical abnormalities were documented. However, the anatomical defect is readily visible when the child is placed in a frog leg position without any traction on the genital tissues (Figure 2).

## Conclusion

This case demonstrates the need for complete inspection of the genitals and anus at birth. While an investigation by child protection agencies was warranted in this case, further information helpful to determining whether and when trauma occurred is unlikely. In this case, a child protection report and investigation, as well as complete testing for sexually transmitted diseases, were conducted due to the hymenal finding. If any anogenital anatomical variants are observed at birth, they should be documented, and the family should be provided with a letter describing the anatomical variant. Such documentation may be helpful for future clinical visits to ensure that the findings are not misattributed to trauma. Identifying anomalies at birth facilitates appropriate assessment and management by other subspecialties, including genetics and surgery.
